# Professor Bartlett

**Published:** 1851-05

**Authors:** 


### PROFESSOR BARTLETT.

This distinguished teacher has resigned the chair he accepted in the New York School last fall. We hope, however, that his valuable talents will not long remain idle, and that his next appointment will prove more agreeable and prosperous than the preceding one did. He is worthy of the highest consideration as a teacher, and we hope to hear soon, that he has a position at once gratifying to him and useful to the profession.

We find the following announcement in the *New Hampshire Journal of Medicine*, for April: "At home changes are being made in professional chairs. Perhaps the most important and the most significant as yet is that Professors Gross and Bartlett leave their chairs in the New York University. This is hardly unexpected by those who are at all acquainted with New York medical polemics—which, by the way, is one of the most curious chapters that we have ever studied. We suspect that it is better understood abroad than the profession in that city are aware." B.

